# Environmental hazard of tick-borne diseases in urban and peri-urban sites in an endemic area of eastern France

**DOI:** 10.1051/parasite/2026043

**Published:** 2026-07-29

**Authors:** Joke Stynen, Cathy Barthel, Armand Maul, Laurine Villeneuve, Nathalie Boulanger

**Affiliations:** 1 UR3073: PHAVI: Borrelia Group, Institute of Bacteriology, University of Strasbourg 67000 Strasbourg France; 2 University of Lorraine, CNRS, LIEC 57000 Metz France; 3 French National Reference Center for Borrelia, Hôpitaux Universitaires 67000 Strasbourg France

**Keywords:** Ecosystem, Greening, *Ixodes ricinus*, Urbanization, Lyme, Acarological hazard

## Abstract

The occurrence of the *Ixodes ricinus* tick in forested and rural areas is well documented across Europe; however, its presence in urban environments remains less investigated. This study focused on the city of Strasbourg, located in eastern France, a region endemic for ticks and tick-borne diseases, to identify high-risk ecosystems along an urbanization gradient. Questing ticks were collected from eight sites: four urban parks, a botanical garden, and three peri-urban forests. A total of 880 ticks were collected, and 657 nymphs were analyzed for the presence of the pathogens *Borrelia burgdorferi* sensu lato, *Anaplasma phagocytophilum*, and *Neoehrlichia mikurensis* using molecular methods. Regularly maintained urban parks presented a low risk of tick bites or tick-borne bacterial infections, including Lyme borreliosis, anaplasmosis, and neoehrlichiosis. In contrast, peri-urban alluvial forests exhibited a high risk of tick bites and potentially infectious diseases, with a mean nymph density of 21.16 (range 18.25–24.75) per 100 m^2^ and a mean density of infected nymph of 7.00 (range 4.93–10.21) per 100 m^2^ for the three pathogens investigated. Owing to their high humidity and diverse fauna, alluvial forests provide particularly favorable microhabitats for *Ixodes* ticks and the circulation of their associated pathogens. In the context of climate change and increasing urban greening initiatives, greener cities may inadvertently increase the risk of tick-borne diseases in urban environments. Therefore, implementing appropriate park and forest management strategies, together with public awareness campaigns, is essential to reduce acarological risk.

## Abstract (fr)

La présence de la tique *Ixodes ricinus* dans les zones forestières et rurales est bien documentée à travers l'Europe ; toutefois, sa présence en milieu urbain a été moins étudiée. Cette étude s'est concentrée sur la ville de Strasbourg, située dans l'est de la France, une région endémique pour les tiques et les maladies qu'elles transmettent, afin d'identifier les écosystèmes à haut risque le long d'un gradient d'urbanisation. Des tiques en phase de quête ont été collectées sur huit sites : quatre parcs urbains, un jardin botanique et trois forêts périurbaines. Au total, 880 tiques ont été récoltées, et 657 nymphes ont été analysées par des méthodes moléculaires pour détecter la présence des pathogènes *Borrelia burgdorferi* sensu lato, *Anaplasma phagocytophilum* et *Neoehrlichia mikurensis*. Les parcs urbains régulièrement entretenus présentaient un faible risque de morsures de tiques ou d'infections bactériennes transmises par ces dernières (borréliose de Lyme, anaplasmose et néoehrlichiose). En revanche, les forêts alluviales périurbaines présentaient un risque élevé de morsures de tiques et de maladies infectieuses, avec une densité moyenne de nymphes de 21,16 (intervalle : 18,25–24,75) pour 100 m^2^ et une densité moyenne de nymphes infectées de 7,00 (intervalle : 4,93–10,21) pour 100 m^2^ pour les trois agents pathogènes étudiés. En raison de leur forte humidité et de la diversité de leur faune, les forêts alluviales offrent des microhabitats particulièrement favorables aux tiques *Ixodes* et à la circulation des agents pathogènes qui leur sont associés. Dans un contexte de changement climatique et de multiplication des initiatives de végétalisation urbaine, le verdissement des villes pourrait involontairement accroître le risque de maladies transmises par les tiques en milieu urbain. Par conséquent, la mise en œuvre de stratégies appropriées de gestion des parcs et des forêts, couplée à des campagnes de sensibilisation du public, est essentielle pour réduire le risque acarologique.

## Introduction

Climate change and land-use transitions, including forest fragmentation, have favored tick expansion in recent decades, increasing the risk of tick bites and tick-borne diseases (TBDs) such as Lyme borreliosis, particularly in the northern hemisphere [4, 15]. In Europe, populations of *Ixodes ricinus* have expanded across a variety of ecosystems as a result of reforestation, increasing urban greening, and the growing abundance of wildlife associated with changes in hunting practices [29, 42, 45]. Consequently, the risk of human TBDs is increasing in urban and suburban environments [53]. A recent review covering 24 European countries reported a mean density of *I. ricinus* in urban green spaces of 6.9 (range 0.1–28.8) ticks per 100 m^2^, with a mean *Borrelia* prevalence of 17.3% (range 3.1–38.1%) [23]. Although urban greening of cities is promoted to mitigate global warming, improve human well-being, and provide recreational opportunities, it may also increase acarological risk [18].

In France, only a few studies have investigated ticks in suburban forests near Paris [37, 38] and within the city of Lyon across different urban ecosystems [40, 49]. In the Lyon region, the prevalence of *B. burgdorferi* s.l. in *Ixodes* ticks was 13.2% [49], and ticks were found to be scarce in urban parks but abundant in peri-urban forests [40]. Previously, we assessed the impact of anthropization on ticks and TBDs across different ecosystems in Strasbourg [7], a city located in eastern France, in a region highly endemic for ticks and TBDs such as Lyme borreliosis, tick-borne encephalitis [58, 69], and anaplasmosis [35]. That study revealed a potential environmental hazard associated with TBDs in certain urban ecosystems, including the Orangerie park and a golf course, due to the presence of several *Borrelia burgdorferi* genospecies and *Borrelia miyamotoi*, the causative agent of relapsing fever [7].

*Ixodes ricinus* is the most important tick species in Europe and is particularly abundant in deciduous and mixed forests. High relative humidity, maintained by leaf litter and forest canopy cover, is essential for its survival [20]. The lifecycle of *I. ricinus* occurs predominantly in vegetation, with only a small proportion of time devoted to blood feeding on a wide range of mammals, birds, and reptiles [20]. Following oviposition by engorged females, ticks develop through three life stages: larva, nymph, and adult male or female. *Ixodes ricinus* is a generalist ectoparasite [28]. Larvae generally feed on small vertebrates such as wood mice (*Apodemus sylvaticus*) and bank voles (*Myodes glareolus*), whereas nymphs parasitize a broader range of hosts, including birds, medium-sized mammals (e.g., hedgehogs, foxes, and hares), and roe deer (*Capreolus capreolus*). Adult females typically feed on large mammals such as deer [33]. Deer play a crucial role in maintaining tick populations [13, 19, 45]; however, alternative hosts such as foxes and hedgehogs may also be fed upon by female ticks in urban parks [20, 70]. In addition, birds contribute to the dispersal of ticks across landscapes [27, 72]. Humans are accidental hosts and are usually exposed when entering habitats where ticks occur, such as wooded or grassy areas.

Among the microorganisms potentially transmitted by *I. ricinus*, *Borrelia burgdorferi* sensu lato (s.l.) is the most important because it is the causative agent of Lyme borreliosis [4, 64]. Other bacterial pathogens transmitted by *I. ricinus* include *Anaplasma phagocytophilum* and *Neoehrlichia mikurensis* [30, 60]. TBDs are zoonoses involving a variety of vertebrate hosts that participate in pathogen maintenance and transmission [4]. For *B. burgdorferi* s.l., the principal reservoir hosts are rodents (e.g., mice, voles, and shrews) and birds (e.g., blackbirds and great tits). Roe deer, not a reservoir-competent for *Borrelia* [20], constitute an important reservoir host for *Anaplasma* [16] and *Babesia venatorum* (formerly *Babesia* EU1) [2]. However, a recent study based on the analysis of remnant blood meals in ticks from urban and forested areas in Finland demonstrated that host importance differs between ecosystem types. In particular, squirrels, leporids [62], and blackbirds [70] appear to be more important hosts in urban environments, whereas roe deer and voles play a more prominent role in forest ecosystems [62].

In the present study, we sampled ticks along an urbanization gradient extending from north to south across Strasbourg. Eight sites were selected, including the university’s botanical garden and several urban parks and peri-urban forests. During the peak period of tick activity, from March to June 2024, we investigated the abundance of questing ticks and the occurrence of three tick-borne bacterial pathogens: *B. burgdorferi* s.l., *A. phagocytophilum*, and *N. mikurensis*. These pathogens are known to occur in eastern France and are responsible for human clinical cases [6, 35, 58]. The main objective of this study was to assess variations in the density of nymphs (DON), density of infected nymphs (DIN), and nymphal infection prevalence (NIP) for the three potentially pathogenic microorganisms along the urbanization gradient and throughout the sampling period.

## Materials and methods

### Study area, sampling sites, and tick collection

**Figure 1:**
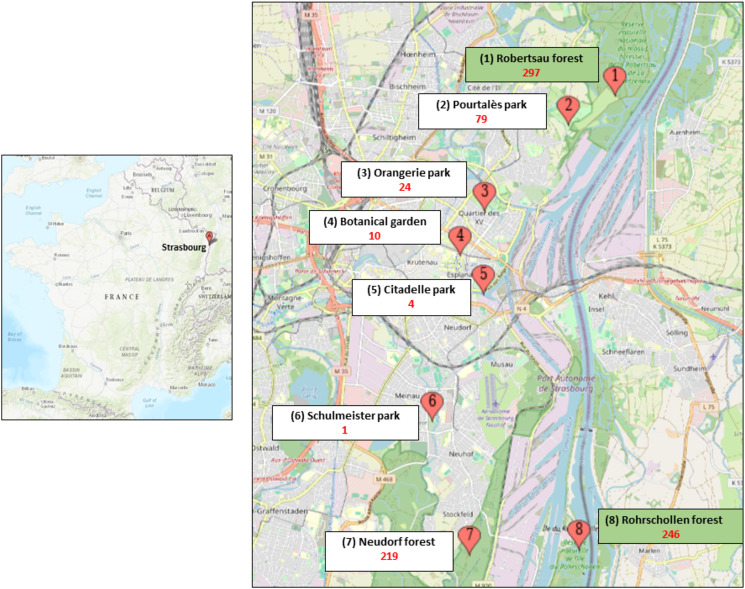
Tick collection sites along the urban gradient of Strasbourg. Total collected nymphs: 880. Total tested nymphs: 657. Red numbers correspond to collected ticks. Green rectangles indicate alluvial forests.

Eight urban and peri-urban sites in Strasbourg, eastern France, were sampled monthly in 2024 (GPS coordinates: 48.5734, 7.7521). Strasbourg has a population of approximately 272,100 and covers an area of 78.26 km^2^. Its altitude ranges from 132 m to 151 m above sea level. The city is surrounded by alluvial forests that have undergone various sylvatic management practices but still contain old-growth stands and areas that have remained unmanaged since 1984. Study sites 1 and 8 were alluvial forests; site 7 was an ancient alluvial forest; and the remaining sites were urban parks, except for site 4, which was a fenced botanical garden belonging to the university (Fig. 1). All the sites are regularly visited by both professionals and the public for recreational and educational activities.

The ticks were collected without handling any vertebrates. Questing ticks were sampled during the peak activity period (March to June 2024) by dragging a 1 m^2^ white cloth over the vegetation along a 300 m^2^ transect at each study site. The cloth was inspected every 10 m^2^, and successive 10-m^2^ transects were separated by 20–30 m to ensure random sampling. Ticks (adults and nymphs) were collected using tweezers, transferred alive into collection tubes, transported to the laboratory, and stored at −20 °C until analysis. At each site, the thirty 10-m^2^ transects were grouped into three 100-m^2^ sections for subsequent analyses.

### DNA extraction and detection of bacterial pathogens by PCR

Up to 60 nymphs per site were randomly selected and analyzed individually. DNA was extracted using the ammonium hydroxide–based method [21, 52]. To detect *B. burgdorferi* s.l. DNA, an initial amplification via real-time PCR was performed to target the conserved region of the *flagellin B* gene, using one primer pair and two Taqman^®^ probes. Amplification and data analysis were carried out using a CFX OPUS96 BIORAD Instrument.

For *Borrelia* genotyping, a second real-time PCR typing assay was performed on all positive samples to identify *B. burgdorferi* s.l. genospecies, using the same primers together with species-specific fluorescent hybridization probes (10 FRET probes and one TaqMan^®^ probe). These probes specifically target *B. burgdorferi* s.s., *B. garinii*/*B. bavariensis*, *B. afzelii*, *B. valaisiana*, and *B. lusitaniae*. Following amplification, the PCR products were subjected to a gradual increase in temperature, and the melting temperature (*T*_m_) of each PCR product/FRET probe duplex was determined. For each genospecies and its probe, the *T*_m_ was specific and enabled species identification [7].

For *A. phagocytophilum*, the *major surface protein 4* (*msp4*) gene was targeted [35]. For *N. mikurensis,* a previously described RT-qPCR assay targeting the *GroEl* gene was used [31]. Positive controls (5 μL *B. japonica* DNA; 5 μL of DNA from tick positive for *Anaplasma* or *B. miyamotoi*) and negative controls (reagents with 5 μL of H_2_O instead of DNA) were included in each PCR run to verify the reaction specificity and detect potential contamination. All PCR assays were performed using a BioRad Opus 384 System.

### MALDI-ToF-MS for tick species identification

Collected ticks were identified by matrix-assisted laser desorption–ionization time-of-flight mass spectrometry (MALDI-TOF-MS) [8]. Briefly, four legs from each adult or nymph tick were analyzed using the same protocol and settings as previously described [5]. A log score value of at least 1.8 was required for reliable species identification, with a minimum difference of 0.2 between the highest and second-highest species match scores.

### Statistical analysis

To assess the risk of acquiring TBDs, we first determined the density of nymphs per 100 m^2^ (DON) and then the nymphal infection prevalence (NIP). We then calculated the density of infected nymphs per 100 m^2^ (DIN) by multiplying DON by NIP, thereby estimating the acarological hazard [25].

Statistical analyses were performed using R software (version 4.2.1) to investigate spatial and temporal variations in nymph density and infection prevalence, and to identify ecosystems exhibiting differences in DON, DIN, and NIP. The experimental design was a nested experiment with three nested factors: sites (eight levels), months (four levels), and replicates (three levels). The non-parametric Friedman test with months (sites) treated as blocks, was used to assess significant differences in DON and DIN among the eight sites and across the four sampling months. Kendall’s rank correlation coefficient was used to evaluate the monotonic relationship between the numbers of infected and tested nymphs. Fisher’s exact test was applied to compare nymphal infection prevalence across sites and months, as well as the relative proportions of the three potentially pathogenic microorganisms among infected nymphs according to site and month.

Generalized linear models (GLMs) were also considered to examine the effects of site and month on DON, DIN, and NIP. DON and DIN were modeled using negative binomial regression with a log-linear link function, whereas NIP was modeled using logistic regression. Separate models were fitted for each pathogen and for all pathogens combined, with site and month included as explanatory variables. Both the non-parametric analyses and the GLMs yielded consistent results. To avoid unnecessary redundancy, we chose to present only the results of the non-parametric analyses in the main text.

This decision was motivated by the characteristics of the dataset, namely the relatively small sample size and the absence of ecological and environmental covariates. Of note, fitting GLMs that include both site and month as explanatory variables may be ambitious given the limited number of observations. Furthermore, models of this kind would be more informative if they incorporated environmental variables known to influence tick abundance such as vegetation type, humidity, and temperature, particularly because sampling conditions may vary substantially among sampling dates. In addition, non-parametric methods, which are characterized by their statistical robustness, perhaps better reflect the scope of this work and are, in fact, largely sufficient to address our research objectives. However, the results of the GLMs are provided in the Supplementary material (Supplementary File 3).

## Results

### Density of *Ixodes* nymphs (DON) across different sites according to ecosystem

A total of 880 ticks were collected over four months in 2024 from eight sites in and around Strasbourg with varying urbanization levels (Supplementary Table S1). Mass spectrometry was used to identify the ticks, as this technique is now a reliable method for identifying different arthropod vectors at the species level [5, 59]. It revealed that 87.3% of randomly tested ticks were *I. ricinus*, and 12.7% were *Ixodes frontalis*. *Ixodes frontalis* was primarily detected in the botanical garden around a bamboo plantation, accounting for 63.6% of ticks collected there. At the remaining sites, only 3.3% of collected ticks were *I. frontalis* (data not shown).

Density of nymph (DON) varied by site and month (Table 1). Statistical analysis revealed varying DONs across the eight sites (Friedman test, *p* < 0.02), regardless of the value given to the missing datum (botanical garden, March).

**Table 1:** Density of nymph (DON) per 100 m^2^ for different sites from March to June 2024.

Month/Site	March	April	May	June	Mean (Standard deviation)
1-Robertsau forest (N)	7.67	26.00	51.67	13.67	**24.75 (19.50)**
2-Pourtalès park (N)	1.00	2.33	6.33	16.67	**6.58 (7.09)**
3-Orangerie park (C)	6.33	0.33	0.00	1.33	**2.00 (2.94)**
4-Botanical garden (C)	*	1.00	1.67	0.67	**1.11 (0.51)**
5-Citadelle park (C)	0.67	0.33	0.33	0.00	**0.33 (0.27)**
6-Schulmeister park (C)	0.00	0.00	0.33	0.00	**0.08 (0.17)**
7-Neuhof forest (S)	19.00	13.67	11.67	28.67	**18.25 (7.60)**
8-Rohrschollen forest (S)	26.67	41.00	6.67	7.67	**20.50 (16.47)**
Mean	8.76 (10.29)	10.54 (15.37)	9.83 (17.40)	8.54 (10.41)	Overall mean 9.46 (13.15)

*No data. N: North, C: City; S: South.

The “site” effect showed that DON was lowest in the city center (mean DON ranging from 0.08/100 m^2^ to 2.00/100 m^2^; SD = 0.17 and 2.94, respectively), whereas it rapidly increased with increasing distance from densely built-up urban areas, both to the north (Pourtalès park: mean = 6.58; SD = 7.09; Robertsau forest: mean = 24.75; SD = 19.50) and the south (Neudorf forest: mean = 18.25; SD = 7.60; Rohrschollen forest: mean = 20.5; SD = 16.47) (Table 1).

Three distinct zones were identified based on nymph abundance: northern sites: Robertsau forest and Pourtalès park, central urban sites: Orangerie park, botanical garden, Citadelle park, and Schulmeister park, and southern sites: Neudorf and Rohrschollen forests. No significant differences in DON were observed among the four months (Friedman test, *p* > 0.90), although the highest DON was recorded in May 2024 in Robertsau forest (51.67/100 m^2^; SD = 6.66) (Table 1).

### Nymphal infection prevalence (NIP)

**Figure 2:**
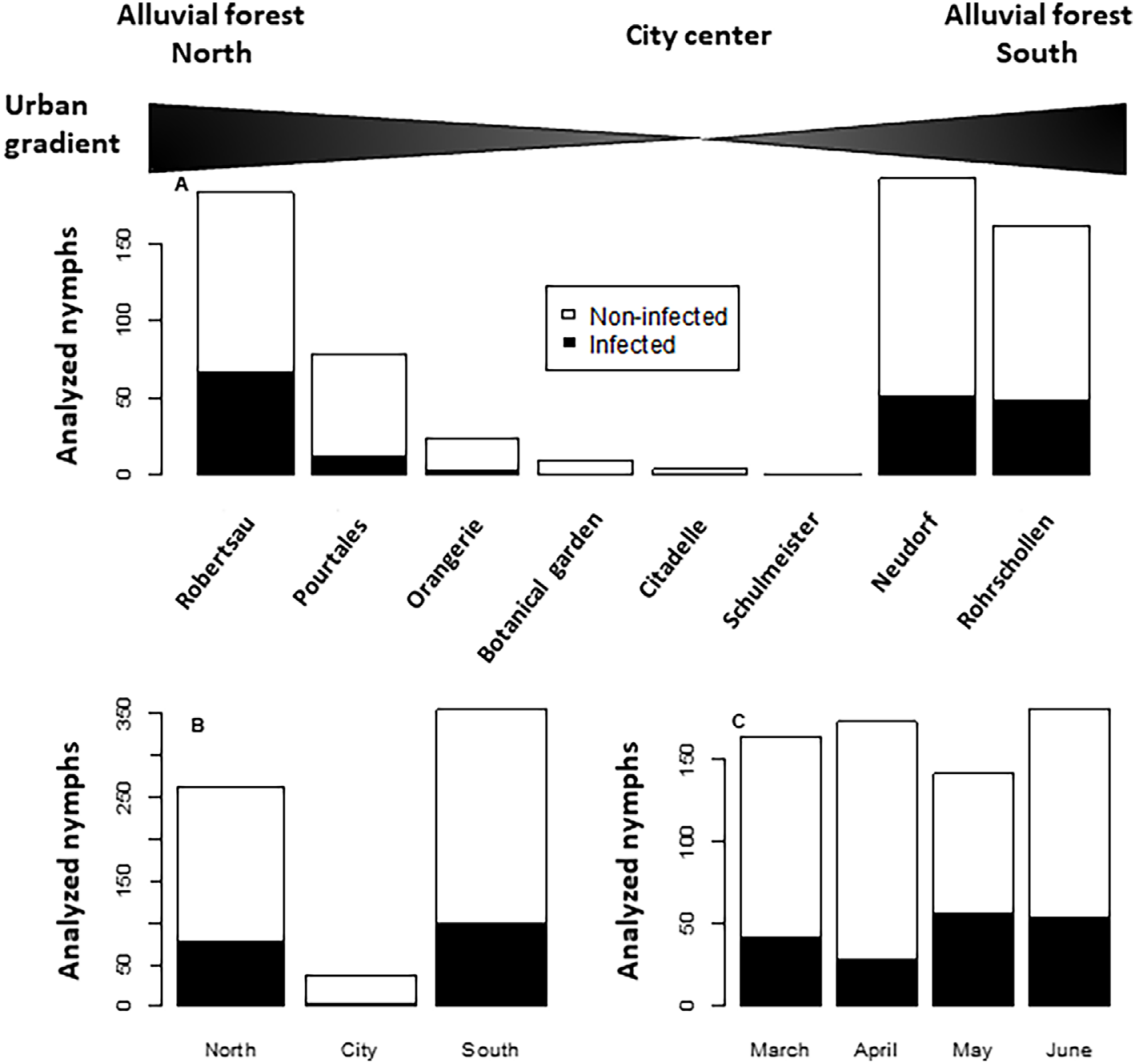
(A) Number of infected and uninfected nymphs at different sites, (B) within the three zones (ecosystems): north, city, and south, (C) over the course of the months.

We observed a strong monotonic relationship between the numbers of infected and analyzed nymphs (Kendall’s *τ* = 0.794, *p* < 10^−4^) (Supplementary Figure S1). The overall NIP was estimated at 27.7% (95% CI: 24.3–31.3). The NIP values significantly differed across the eight sites (Fisher’s exact test, *p* = 0.003) and the three zones (*p* = 0.022) (Figs. 2A and 2B). In the north, NIP was significantly higher in Robertsau forest (36.4%; 95% CI: 29.5–43.9, 67/184) than in Pourtalès park (15.2%; 95% CI: 8.4–25.4, 12/79) (Supplementary Table S2a). Furthermore, the NIP was lower in city parks (10.3%; 95% CI: 4.1–23.6, 4/39) than in alluvial forests. In addition, the NIP in the alluvial forests of the northern sites (30.0%; 95% CI: 24.8–35.8, 79/263) and the southern sites (27.9%; 95% CI: 23.5–32.8, 99/355) was comparable (Supplementary Table S2b). The NIP varied significantly across the four months (*p* < 0.001), with the highest being in May (40.4%; 95% CI: 32.3–49.0, 57/141) and the lowest being in April (16.8%; 95% CI: 11.7–23.4, 29/173) (Supplementary Table S2c).

The prevalence of the three potentially pathogenic microorganisms, *B. burgdorferi* s.l., *A. phagocytophilum*, and *N. mikurensis* was determined (Table 2). Among the 657 nymphs analyzed, 182 were infected (27.7%; 95% CI: 24.3–31.3). Because 31 nymphs were co-infected (4.7%; 95% CI: 3.2–6.7, 31/657), a total of 214 infections were recorded. Co-infections consisted of 26 cases involving *B. afzelii* and *N. mikurensis*, four involving *B. burgdorferi* s.l. and *A. phagocytophilum*, and one triple co-infection involving all three microorganisms (Robertsau forest, May 2024).

**Table 2:** Bacterial prevalence on the *Ixodes ricinus* nymphs.

	No. positive / 657 analyzed nymphs	% positive	95% CI
Infected nymphs (overall)	182	27.7	24.3–31.3
*Borrelia burgdorferi* s.l.	127	19.3	16.5–22.5
*Anaplasma phagocytophilum*	10	1.5	0.8–2.8
*Neoehrlichia mikurensis*	77	11.7	9.5–14.4
Co-infections*	31	4.7	3.2–6.7

*Co-infections / co-occurrences: 26 *B. afzelii* + *N. mikurensis*; 1 *B. garinii* + *A. phagocytophilum*; 1 *B. lusitaniae + A. phagocytophilum*; 1 *B. valaisiana + A. phagocytophilum*; 1 *B. burgdorferi* s.s. + *A. phagocytophilum*; 1 triple infection: *B. afzelii + A. phagocytophilum + N. mikurensis.*

**Figure 3:**
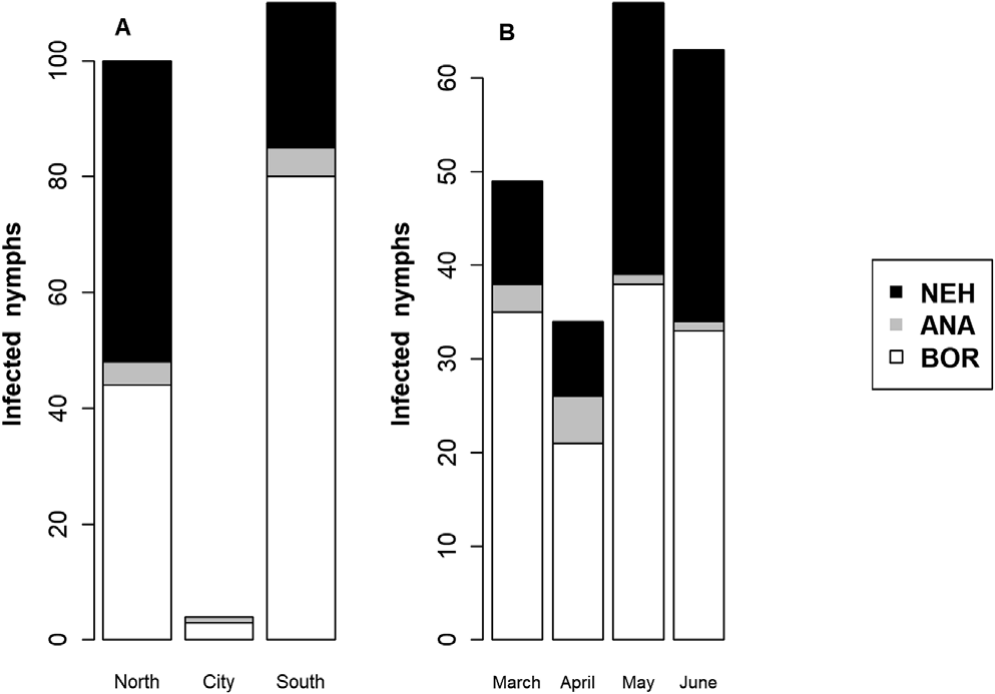
Abundance of the three potentially pathogenic bacteria in infected nymphs, (A) across the three areas (ecosystems) – namely north, city, and south, and (B) over the course of several months. Abbreviations: BOR, *Borrelia burgdorferi* sensu lato; ANA, *Anaplasma phagocytophilum*, and NEH, *Neoehrlichia mikurensis*.

The relative proportions of the three bacteria in infected nymphs varied across sites (*p* < 0.001), zones (*p* < 10^−4^), and months (*p* = 0.006) (Fig. 3 and Supplementary Table S3). The proportion of *B. burgdorferi* s.l. was low at the northern sites (44.0%; 95% CI: 34.2–54.3, 44/100) and high at the southern sites (72.7%; 95% CI: 63.3–80.6, 80/110). *Neoehrlichia mikurensis* showed the opposite trend (north: 52.0%; 95% CI: 41.8–62.0, 52/100; south: 22.7%; 95% CI: 15.5–31.9, 25/110). The proportion of *A. phagocytophilum* in infected nymphs was relatively high in April (14.7%, 95% CI: 5.5–31.8, 5/34).

### Acarological hazard and presence of *Borrelia* genospecies in ecosystems

The acarological risk was defined as the likelihood of encountering infected ticks in a given area. This risk was quantified using the DON and NIP. It refers to the density of infected nymphs (DIN). Since we had no data on human exposure, we used the term “acarological hazard”. DIN varied among sites and sampling months (Table 3). Significant differences in DIN were observed among the eight sites (Friedman test, *p* < 0.01), whereas no significant differences were detected among the four sampling months (*p* > 0.40). The highest DIN was recorded in May in Robertsau alluvial forest (28.42 infected nymphs/100 m^2^) (Table 3). Mean DIN values were highest in the alluvial forests located north and south of Strasbourg (Robertsau: 10.21; Neudorf: 4.93; Rohrschollen: 5.88). No infected ticks were detected in city parks (Citadelle and Schulmeister). In Orangerie park, only three ticks infected with *Borrelia* were detected, whereas a single tick infected with *A. phagocytophilum* was found in the botanical garden during the study period (Supplementary Table S3a).

**Table 3:** Density of the infected nymphs (DIN)/100 m^2^ at different sites from March 2024 to June 2024.

Month/Site	March	April	May	June	Mean (Standard deviation)
1-Robertsau forest	2.67	4.77	28.42	5.00	10.21 (12.18)
2-Pourtalès park	0.00	0.00	1.00	3.00	1.00 (1.41)
3-Orangerie park	0.67	0.00	0.00	0.33	0.25 (0.32)
4-Botanical garden	*	0.00	0.00	0.33	0.11 (0.19)
5-Citadelle park	0.00	0.00	0.00	0.00	0.00 (0.00)
6-Schulmeister park	0.00	0.00	0.00	0.00	0.00 (0.00)
7-Neuhof forest	4.33	1.00	5.33	9.08	4.93 (3.32)
8-Rohrschollen forest	8.59	10.25	1.67	3.00	5.88 (4.18)
Mean	2.32 (3.22)	2.00 (3.72)	4.55 (9.81)	2.59 (3.20)	Overall mean 2.88 (5.59)

*No data.

**Figure 4:**
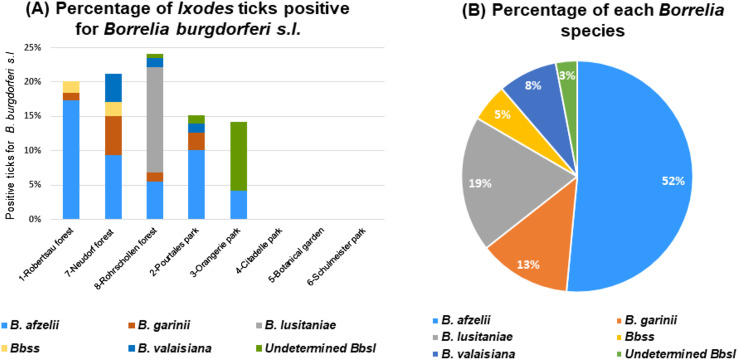
(A) *B. burgdorferi* sensu lato infection prevalence per site in 2024, (B) overall prevalence of *B. burgdorferi* s.l. (Bbsl) species in 2024. Bbss: *Borrelia burgdorferi* sensu stricto. 1, 7, 8: alluvial forests; 2, 3: city parks with connectivity to a forest; 4, 5, 6: city parks and botanical garden.

*Borrelia burgdorferi* s.l. is the most important pathogen for humans in the northern temperate zones [65], particularly in France [57]. We identified human-pathogenic *Borrelia* genospecies. *Borrelia afzelii* was the most represented genospecies at all sites (52%), except in the city center (Citadelle, Schulmeister, and botanical garden), followed by the bird-associated species such as *B. garinii* (13%) and *B. valaisiana* (19%) (Fig. 4). Notably, a significant number of ticks infected with *B. lusitaniae* (8%) was detected in Rohrschollen forest. *Borrelia* species exhibited co-infection with other microorganisms at a rate of 4.7%. The most frequent association was *N. mikurensis*–*B. burgdorferi* s.l. (Table 2).

Overall, no significant temporal variation in DON and DIN was observed during the four-month sampling period (Friedman test, *p* > 0.90 and *p* > 0.40, respectively). These statistical results may appear inconsistent with the data presented in Tables 1 and 3, which show noticeable month-to-month differences in the mean values of DON and DIN. This apparent discrepancy can be explained both by the high variability among sites and the limited statistical power of the Friedman test.

## Discussion

The spread of *Ixodes* ticks in rural ecosystems has raised concerns about their increasing prevalence in urban and peri-urban areas across Europe. While urban greening, promoted to combat climate change and improve human well-being, creates novel ecosystems that favor ticks; it also increases the risk of tick-borne diseases (TBDs) for urban residents [15, 26, 67]. Additionally, efforts to enhance biodiversity through urban greening, the protection of certain natural areas, and changes in forestry practices are promoting connectivity between the ecosystems and the movement of wildlife.

The Strasbourg region, located in the Alsace Plain, has undergone significant landscape changes in recent decades, shifting from mixed cropping to maize monoculture, which has led to landscape fragmentation. The city of Strasbourg, named French Capital of Biodiversity in 2014 under the theme “Urban Agriculture and Biodiversity”, has preserved numerous natural areas, including three Natura 2000 sites, and manages 430 ha of green spaces. Its local climate plan includes 13 objectives, such as developing a green belt as a plant shield. This study was conducted as part of the European URBACT project (Strasbourg, “One Health 4 Cities”), which aims to revitalize a small watercourse connected to the Rhine River system and raise awareness among local residents.

A previous survey in Strasbourg (2018–2019) revealed that certain parks and peri-urban forested areas were highly conducive to *Ixodes* and *Dermacentor* tick populations [1, 7]. The present study highlights that alluvial forests around Strasbourg are also highly favorable ecosystems for these ticks. We focused on *Ixodes* nymphs, as they are the most common life stage found biting humans [34]. In this study, *Ixodes* nymphs were significantly infected with *B. burgdorferi* s.l. (19.3%), *A. phagocytophilum* (1.5%), and *N. mikurensis* (11.7%). The abundance of wildlife in Strasbourg enables the circulation of these bacteria, whose primary reservoirs are forest rodents [31, 62] and birds [74], as well as squirrels and shrews in urban areas [62]. Parks in central Strasbourg had few ticks. Tick presence in these ecosystems is likely due to specimens introduced by birds, particularly blackbirds (*Turdus merula*), as not all developmental stages were collected [14]. The DIN was zero in two city parks, Citadelle and Schulmeister, likely due to poor understory vegetation and an unfavorable ground microclimate for *Ixodes* ticks and their host community [45]. There is no connectivity between these parks and alluvial forests, a factor known to promote tick presence in cities via the movement of vertebrate hosts [26, 67], creating a metaecosystem [17]. The NIP in alluvial forests (30%) was higher than in central Europe (16.7%) [66]. In other studies conducted in eastern France, lower mean *B. burgdorferi* s.l. infection rates were detected, with NIPs of 12.5% in Moselle and 9.8% in Argonne region [3, 22], both rural areas. The NIP for *N. mikurensis* in Strasbourg (11.7%) was similar to that in Argonne (11.2%). The absence of this bacterium in the city suggests that its preferred reservoirs (mice and voles) are not present [31].

Regarding *Borrelia* genospecies, the most abundant species were *B. afzelii*, *B. valaisiana*, *B. garinii*, *B. lusitaniae*, and *B. burgdorferi* s.s. in descending order. In Europe, *B. afzelii*, whose reservoirs include mice, shrews, and squirrels [62], is generally the most abundant, followed by bird-associated species *B. garinii* and *B. valaisiana* [66]. *Borrelia afzelii*, *B. garinii*, and *B. burgdorferi* s.s. are well-known human pathogens and primarily responsible for Lyme disease in Europe and North America [65]. Notably, *B. lusitaniae*, associated with lizards [44, 74], was detected in Rohrschollen forest. This species is considered a rare or potential cause of Lyme borreliosis [12, 68], with less typical clinical manifestations. Its presence is increasingly documented in other European regions [9, 10].

Bacterial co-infection in *Ixodes* nymphs was 4.7%. In a three-year study in a forest near Paris, a prevalence of 1.3% was observed after testing for 31 pathogens [37]. The most common co-infection was *N. mikurensis* with *B. afzelii*, which has also been observed elsewhere in Europe [36]. Rodents (*Myodes*, *Apodemus*, or *Microtus*) are suspected as the main hosts for this association [11]. *Anaplasma phagocytophilum* was associated with *B. garinii* and *B. valaisiana*, both of which are commonly linked to birds. Blackbirds (*Turdus merula*) are good reservoirs for these pathogens in urban ecosystems [70, 73]. The acarological risk in Strasbourg and the Alsace region cannot be overlooked, as human cases of TBDs are regularly reported, including anaplasmosis [35], neoehrlichiosis [6], babesiosis [39], and tick-borne encephalitis [69]. Screening for the tick-borne encephalitis virus in ticks would be particularly worthwhile at this time. Interestingly, the first human case was reported in 1968 in Strasbourg [69]. A study conducted from 1970 to 1974 on ticks and rodents revealed that the virus was circulating in the Neudorf Forest (site 7 of the present study) [47]. France made the disease a notifiable disease in 2021 and has noted a resurgence of cases throughout the Grand-Est region [56].

We did not survey private gardens, which may be important at-risk environments for tick bites and TBDs [43, 51, 54]. However, we assessed one community garden and found no ticks. Private gardens in the Netherlands [43], Belgium [54], and Germany [51] have been studied for potential acarological risks. Depending on their location, these gardens may promote tick development if near forests, with abundant leaf litter, hedges, composting areas, and certain hosts [41]. Among the tick species identified in these studies, *I. ricinus*, a generalist tick that feeds on more than 300 host species [28], was the most abundant, followed by *I. frontalis,* which shows a strong preference for birds [48] and *I. hexagonus* which uses hedgehogs as hosts [54]*.* In this study, we collected mainly *I. ricinus* ticks, and very few *I. frontalis* ticks in the botanical garden and a few in the alluvial forests.

In France, few studies have focused on the risk of ticks and associated pathogens in urban and peri-urban ecosystems [37, 38, 40, 50, 71]. Several European studies have investigated tick presence in cities [9, 14, 24, 32, 40, 55, 61], and the detection of potentially pathogenic microorganisms in these ticks has highlighted a potential acarological risk [9, 32, 46, 53, 61]. Vegetation and woodland restorations aim to limit climate change and promote biodiversity and human well-being in urban ecosystems [45]. However, these changes in land use, especially in TBD-endemic areas, increasingly expose communities to previously unknown risks. The presence of diverse hosts in cities allows the development and survival of *Ixodes* ticks, especially in parks connected to forested ecosystems.

It is crucial to inform local municipalities about the need for ecosystem management and to propose preventive measures for professionals and the public [18]. Concerted actions are needed, including control strategies targeting wildlife, ecosystem management, prevention measures, public awareness campaigns, and accurate medical settings for diagnosing TBDs [4, 63]. Although limited to Strasbourg and one season, this study provides a detailed overview of the spatial distribution of the tick-borne disease hazard in a city located within a region highly endemic for TBDs.

## Figures and Tables

**Figure 5:**
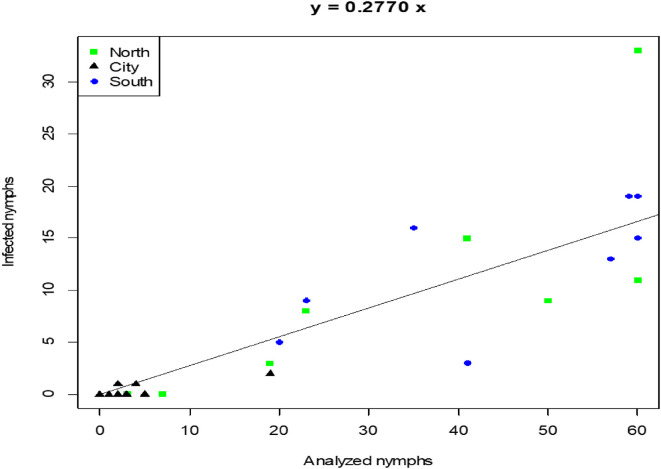
*Supplementary Figure S1*: Infected nymphs *versus* analyzed nymphs within the three zones (ecosystems) examined.

## Data Availability

All data and materials are available upon request.

## Supplementary material

*Supplementary File 1*: Study sites. *Supplementary File 2*: Animals present at the various sites according to GEPMA (Groupe d’Étude et de Protection des Mammifères d’Alsace). *Supplementary File 3*: Regression analyses Generalized linear models (GLMs). Negative binomial regression for nymphal abundance (DON). Logistic regression for nymphal infection prevalence (NIP). *Supplementary Table S1*: Number of *Ixodes ricinus* nymphs collected at the sites from March to June 2024. Counts for each triplicate and total per site and month.

*Supplementary Table S2*: Nymphal infection prevalence (NIP). Table S2a: Nymphal infection prevalence (NIP) for the 8 sites. Table S2b: Nymphal infection prevalence (NIP) for the 3 zones (ecosystems). Table S2c: Nymphal infection prevalence (NIP) for the 4 months. *Supplementary Table S3*: Bacterial pathogens. Tested bacteria: BOR: *Borrelia burgdorferi* sensu lato, ANA: *Anaplasma phagocytophilum*, NEH: *Neoehrlichia mikurensis.* Table S3a: Pathogen abundance for each bacterium *versus* sites. Table S3b: Bacteria abundance *versus* zones (after grouping of sites). Table S3c: Bacteria abundance *versus* months. The supplementary material of this article is available at https://www.parasite-journal.org/10.1051/parasite/2026043/olm.
