# Supplementary material for: Environmental hazard of tick-borne diseases in urban and peri-urban sites in an endemic area of eastern France
Source: Parasite. 2026 Jul 29;33:40. doi: 10.1051/parasite/2026043 (PMC13427044; doi:10.1051/parasite/2026043)

Supplementary File 1: Study sites

(1) Robertsau forest - North	(2) Pourtalès park - North
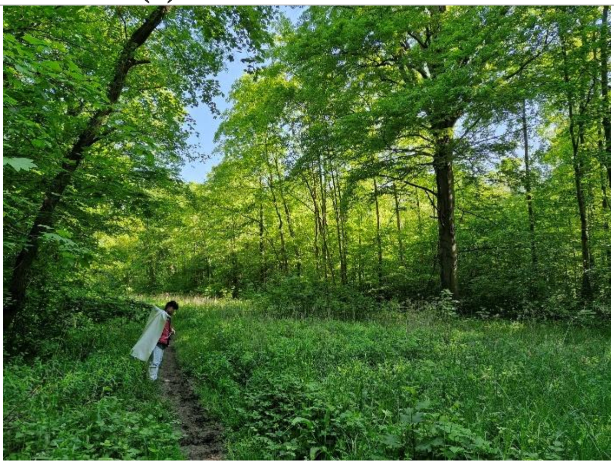	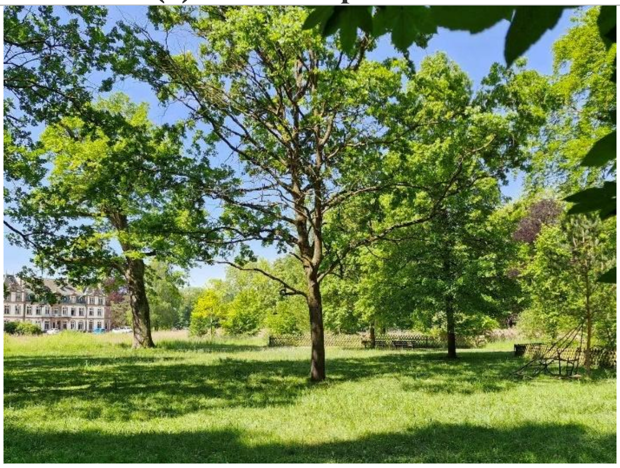
(3) Orangerie park	(4) Botanical garden
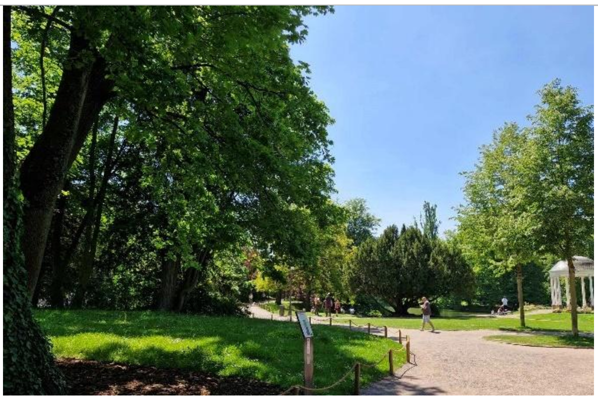	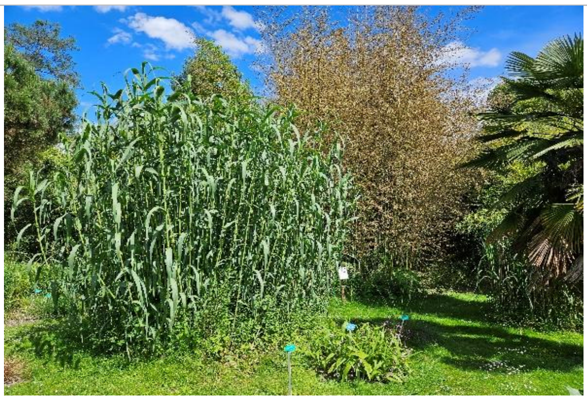
(5) Citadelle park	(6) Schulmeister park
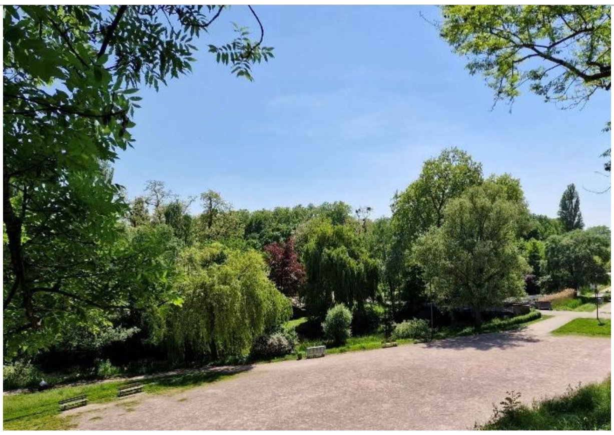	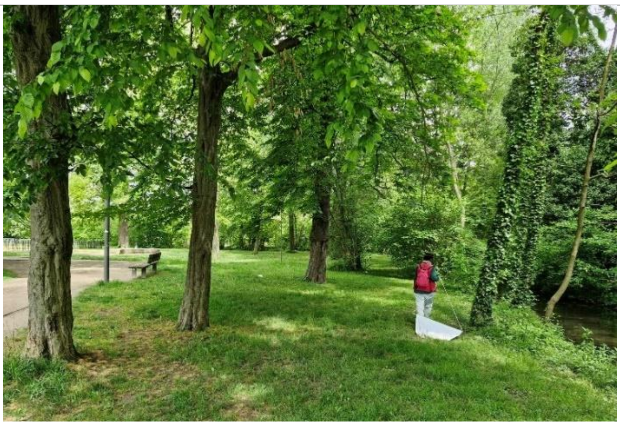
(7) Neudorf forest (South)	(8) Rohrschollen forest (South)

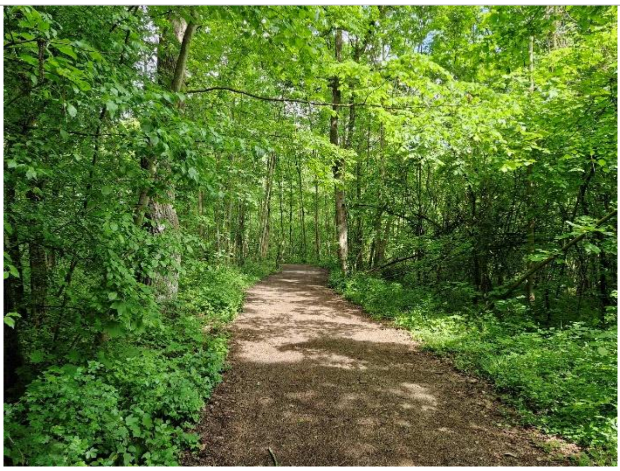

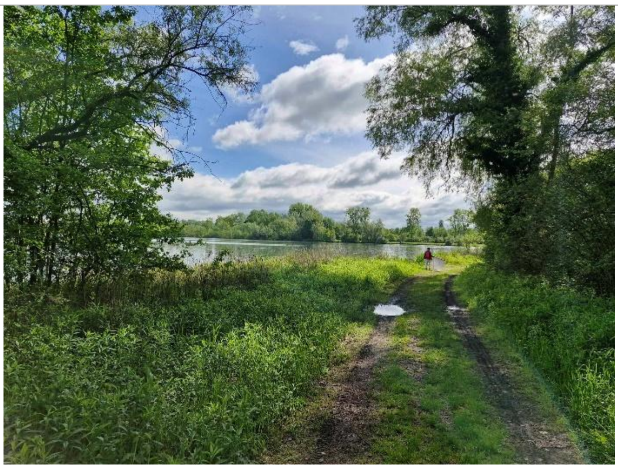

Supplement: Supplementary file 1 — Study sites. [file parasite-33-40-s1.pdf]
