# Supplementary material for: Environmental hazard of tick-borne diseases in urban and peri-urban sites in an endemic area of eastern France
Source: Parasite. 2026 Jul 29;33:40. doi: 10.1051/parasite/2026043 (PMC13427044; doi:10.1051/parasite/2026043)
Supplement: Supplementary file 2 — Animals present at the various sites according to GEPMA (Groupe d’Étude et de Protection des Mammifères d’Alsace). [file parasite-33-40-s2.pdf]

Supplementary File 2: Animals present at the various sites according to GEPMA (Groupe d’Étude et de Protection des Mammifères d’Alsace) - https://gepma.org/

Collection site + coordinates	Animals © BDD Association GEPMA	
	English name	Latin name
**(1) Robertsau Forest**<br>(48.6157, 7.8133)<br>Alluvial forest. Located to the north of Strasbourg; part of the Massif forestier de la Robertsau et de La Wantzenau National Nature Reserve<br>493 hectares	Common pipistrelle	*Pipistrellus pipistrellus*
	Daubenton’s bat	*Myotis daubentonii*
	Roe deer	*Capreolus capreolus*
	Red squirrel	*Sciurus vulgaris*
	Nutria	*Myocastor coypus*
	Wild boar	*Sus scrofa*
	Brown rat	*Rattus norvegicus*
	Bat	*Chiroptera*
	Muskrat	*Ondatra zibethicus*
	European rabbit	*Oryctolagus cuniculus*
	Red fox	*Vulpes vulpes*
	European hedgehog	*Erinaceus europaeus*
	European hare	*Lepus europaeus*
	European badger	*Meles meles*
	Eurasian harvest mouse	*Micromys minutus*
	Undetermined weasel	*Mustela*
	European mole	*Talpa europaea*
	Common vole	*Microtus arvalis*
	Least weasel	*Mustela nivalis*
	Beaver	*Castor fiber*
	Undetermined micromammal	/
	Bank vole	*Myodes glareolus*
	Undetermined field mouse	
	Birds	
**(2) Pourtalès park**<br>(48.6096, 7.7996)<br>Urban park, next to Robertsau forest<br>24 hectares	Red squirrel	*Sciurus vulgaris*
	European hedgehog	*Erinaceus europaeus*
	European red fox	*Vulpes vulpes crucigera*
	Soprano pipistrelle bat	*Pipistrellus pygmaeus*
	Roe deer	*Capreolus capreolus*
	Wild boar	*Sus scrofa*
	Eurasian harvest mouse	*Micromys minutus*
	Brown rat	*Rattus norvegicus*
	Undetermined field mouse	
	Crowned shrew	*Sorex coronatus*
	Greater white-toothed shrew	*Crocidura russula*
	Eurasian pygmy shrew	*Sorex minutus*
	Wood mouse (field mouse)	*Apodemus sylvaticus*
	Common vole	*Microtus arvalis*
	Yellow-necked mouse	*Apodemus flavicollis*
	Field vole	*Microtus agrestis*

	Bank vole<br>Eurasian water shrew<br>Daubenton’s bat<br>European hare<br>House mouse<br>Southwestern/European water vole<br>Least weasel<br>European mole<br>European rabbit	*Myodes glareolus*<br>*Neomys fodiens*<br>*Myotis daubentonii*<br>*Lepus europaeus*<br>*Mus musculus*<br>*Arvicola sapidus/ amphibius*<br><br>*Mustella nivalis*<br>*Talpa europaea*<br>*Oryctolagus cuniculus*
**(3) Orangerie park**<br><br>(48.5924, 7.7747)<br>Urban park<br>Large trees, shrubs, and bushes<br><br>26 hectares	Grey long-eared bats<br>Red squirrel<br>Noctule bat<br>Soprano pipistrelle<br>European hedgehog<br>Common pipistrelle<br>Undetermined bat<br>Bat<br>Kuhl’s pipistrelle bat<br>Brown rat<br>Red fox<br>Nutria	*Plecotus austriacus*<br>*Sciurus vulgaris*<br>*Nyctalus noctula*<br>*Pipistrellus pygmaeus*<br>*Erinaceus europaeus*<br>*Pipistrellus pipistrellus*<br>*Myotis* sp.<br>Chiroptera<br>*Pipistrellus kuhlii*<br>*Rattus norvegicus*<br>*Vulpes vulpes*<br>*Myocastor coypus*
**(4) Botanical garden**<br><br>(48.5836, 7.7666)<br>Part of the university campus and a fenced garden with more than 6,000 plant species and large trees.<br><br>3.5 hectares	Red fox<br>Noctule bat<br>European hedgehog<br>Brown rat<br>Weasel<br>Mice<br>Domestic cat<br>Squirrel	*Vulpes vulpes*<br>*Nyctalus noctule*<br>*Erinaceus europaeus*<br>*Rattus norvegicus*<br>*Mustela* spp.<br>*Rodentia*<br>*Felis catus*<br>*Sciurus vulgaris*
**(5) Citadelle park**<br><br>(48.5758, 7.7745)<br>Large trees, maintained lawns<br><br>12.5 hectares	Daubenton’s bat<br>Noctule bat<br>Bat<br>Beech marten<br>Red fox<br>Red squirrel<br>Nutria<br>Harvest mouse<br>Passerine birds: blackbird, chaffinch, great tit, starling, sparrow, pigeon	*Myotis daubentoniid*<br>*Nyctalus noctule*<br>*Chiroptera*<br>*Martes foina*<br>*Vulpes vulpes*<br>*Sciurus vulgaris*<br>*Myocastor coypus*<br>*Micromys minutus*
**(6) Schulmeister park**	Red fox<br>Noctule bat	*Vulpes vulpes*<br>*Nyctalus noctule*

(48.5505, 7.7594)<br>Large trees, maintained lawns<br><br>7 hectares	European hedgehog<br>Nutria<br>Brown rat<br>Passerine birds: blackbird, chaffinch, great tit, starling, sparrow, pigeon	*Erinaceus europaeus*<br>*Myocastor coypus*<br>*Rattus norvegicus*
**(7) Neudorf forest**<br><br>(48.5236, 7.7704)<br>Ancient alluvial forest. Since 2012, part of the National Nature Reserve of the Strasbourg-Neuhof/Illkirch-Graffenstaden forest massif.<br><br>757 hectares	Beech marten<br>Noctule bat<br>Hazel dormouse<br>Red squirrel<br>Red fox<br>Harvest mouse<br>Roe deer<br>Common pipistrelle<br>Leisler’s bat<br>Soprano pipistrelle<br>Undetermined bat<br>Feral cat<br>Soprano/Kuhl’s/common/ Nathusius’s pipistrelle<br>Garden dormouse<br>Undetermined pipistrelle<br>Wild boar<br>European pine marten<br>Brown rat<br>Nutria<br>European badger<br>European hedgehog<br>Marten/Beech marten<br>European mole<br>Birds	*Martes foina*<br>*Nyctalus noctula*<br>*Muscardinus avellanarius*<br>*Sciurus vulgaris*<br>*Vulpes vulpes*<br>*Micromys minutus*<br>*Capreolus capreolus*<br>*Pipistrellus pipistrellus*<br>*Nyctalus leisleri*<br>*Pipistrellus pygmaeus*<br>*Myotis sp.*<br>*Felis catus*<br>*Pipistrellus pipistrellus /kuhlii/ pygmaeus/nathusii*<br>*Eliomys quercinus*<br>*Pipistrellus*<br>*Sus scrofa*<br>*Martes martes*<br>*Rattus norvegicus*<br>*Myocastor coypus*<br>*Meles meles*<br>*Erinaceus europaeus*<br>*Martes/Martes fiona*<br>*Talpa europaea*
**(8) Rohrschollen forest**<br><br>(48.5247, 7.8026)<br><br>The Rohrschollen Island National Nature Reserve was created in 1997. Located on the Rhine, southeast of Strasbourg and 5.3 km long, 100 to 750 m wide.<br><br>310 hectares	European rabbit<br>Harvest mouse<br>Kuhl’s/Nathusius's pipistrelle<br>Bat<br>Roe deer<br>Nutria<br>Muskrat<br>Wild boar<br>Marten/Beech marten<br>Hazel dormouse<br>Least weasel<br>Red squirrel<br>Red fox<br>Bank vole<br>European badger<br>Undetermined shrew	*Oryctolagus cuniculus*<br>*Micromys minutus*<br>*Pipistrellus kuhlii/nathusii*<br>*Chiroptera*<br>*Capreolus capreolus*<br>*Myocastor coypus*<br>*Ondatra zibethicus*<br>*Sus scrofa*<br>*Martes/Martes fiona*<br>*Muscardinus avellanarius*<br>*Mustela nivalis*<br>*Sciurus vulgaris*<br>*Vulpes vulpes*<br>*Myodes glareolus*<br>*Meles meles*<br>Soricinae

	Numerous birds: 158 bird species and the site is home to more than 4,000 wintering waterfowl, making the nature reserve a first-rate ornithological site. Amphibians and reptiles.	
