# Supplementary material for: Environmental hazard of tick-borne diseases in urban and peri-urban sites in an endemic area of eastern France
Source: Parasite. 2026 Jul 29;33:40. doi: 10.1051/parasite/2026043 (PMC13427044; doi:10.1051/parasite/2026043)
Supplement: Supplementary file 3 — Regression analyses Generalized linear models (GLMs). Negative binomial regression for nymphal abundance (DON). Logistic regression for nymphal infection prevalence (NIP). [file parasite-33-40-s3.pdf]

Supplemental File 3: Regression analyses

Generalized linear models (GLMs)

DON was modeled using negative binomial regression with a log-linear link function. Clearly, the logarithm of the mean number of nymphs was expressed as a linear function of sites and months, considered as explanatory factors. This approach is particularly well-suited for dealing with non-normally distributed, over-dispersed count data. Similarly, we modeled the logit of the proportion of nymphs infected by pathogens (NIP) using logistic regression, again using sites and months as explanatory variables. This approach was adopted for the three pathogens taken individually or together.
The previous models, which belong to the generalized linear model (GLM) family, were fitted to the data using the MASS package in R software. The best-fitting reduced models were determined using a stepwise procedure with backward elimination of variables. This procedure was achieved by minimizing the Akaike Information Criterion (AIC) while ensuring that the effects of the selected variables were statistically significant. A significance level of $\alpha = 0.01$ was chosen to minimize Type I errors in statistical testing.
The structure of the experimental design allows us to model nymph abundance and nymphal infection prevalence as a function of sites and months, and to use the fitted models for both predictive and explanatory purposes.

Negative binomial regression for nymphal abundance (DON)

Negative binomial regression allows for a quantitative assessment of our results; thus, the reduced fitted model describing the abundance of nymphs (DON) as a function of the explanatory variables is as follows.

$$\log_{10} \text{DON} = \begin{cases} 1.348 \text{ Robertsau} \\ 0.773 \text{ Pourtalès} \\ 1.216 \text{ Neuhof} \\ 1.266 \text{ Rohrschollen} \end{cases}$$

The fitted model supported our previous results as it reveals the “Site” effect and shows that the “Month” effect was not statistically significant ($p = 0.95$).
Furthermore, at the 1% level of risk, the regression coefficients associated with the four urbanized sites (i.e., Orangerie, botanical garden, Citadelle, and Schulmeister) were not statistically different from zero and, therefore, not included in the model.
The Table below provides a summary of the negative binomial regression analysis.

Negative binomial regression output for nymphal abundance (DON).

	Estimate	Standard error	z value	Pr(>|z|)
Intercept	0.0458	0.1799	0.2546	0.7992
Robertsau	1.3478	0.2077	6.4898	8.55e-11 **
Pourtalès	0.7727	0.2118	3.6474	0.0003 **

Orangerie	0.2553	0.2244	1.1377	0.2552
Citadelle	-0.5229	0.2994	-1.7465	0.0807
Schulmeister	-1.1250	0.4807	-2.3402	0.0193
Neudorf	1.2155	0.2082	5.8381	5.28e-09 **
Rohrschollen	1.2660	0.2080	6.0870	1.15e-09 **

** significant at the 1% probability level
Dispersion parameter: θ=1.552
Null deviance: 302.302 on 92 degrees of freedom
Residual deviance: 91.299 on 85 degrees of freedom
AIC: 475
Mean Standard Error: 0.336
2 x log-likelihood: -457

Logistic regression for nymphal infection prevalence (NIP)

The logit modeling of the proportion (p) of infected nymphs quantified and showed the ranking of months in terms of NIP.

$$\mathrm{Ln}\left(\frac{\mathrm{p}}{1-\mathrm{p}}\right) = -1.602 + \begin{cases} 1.215 \text{ May} \\ 0.755 \text{ June} \\ 0.544 \text{ March} \end{cases}$$

Modeling the proportions (p) of the three bacteria examined in the infected nymphs yielded the following reduced models:

B. burgdorferi s.l.: $\ln\left(\frac{\mathrm{p}}{1-\mathrm{p}}\right) = -1.979 + \begin{cases} 0.982 \text{ May} \\ 0.683 \text{ March} \end{cases}$

N. mikurensis: $\ln\left(\frac{\mathrm{p}}{1-\mathrm{p}}\right) = -3.026 + \begin{cases} 1.675 \text{ May} \\ 1.376 \text{ June} \end{cases}$

For *A. phagocytophilum*, no model could be fitted due to insufficient data.

The three fitted logistic models presented above highlight the effect of months on the global NIP, as well as on the relative proportions of *B. burgdorferi* s.l., and *N. mikurensis* present in infected nymphs. Sites were not selected and included in these models, although non-parametric tests revealed significant differences between them, both for NIP (Fisher's exact test, $p = 0.003$) and the relative proportions of the three bacteria studied in infected nymphs ($p < 0.001$). This disparity may result from the structure of the available data (i.e., the relatively small number of observations for each site) and/or from the differences in scope and power of the statistical tests performed.
The Tables below present a summary of the logistic regression analyses corresponding to each of the fitted models, for NIP, *B. burgdorferi s.l.*, and *N. mikurensis*, respectively.

Logistic regression output for nymphal infection prevalence (NIP).

	Estimate	Standard error	z value	Pr(>|z|)
Intercept	-1.6025	0.2035	-7.8747	3.45e-15 **
June	0.7552	0.2605	2.8990	0.0037 **
March	0.5444	0.2711	2.0081	0.0446 *
May	1.2148	0.2662	4.5635	5.05e-06 **

** significant at the 1% probability level
Null deviance: 67.550 on 25 degrees of freedom
Residual deviance: 44.764 on 22 degrees of freedom
AIC: 112.2

Logistic regression output for *Borrelia burgdorferi* sensu lato prevalence.

	Estimate	Standard error	z value	Pr(>|z|)
Intercept	-1.9794	0.2328	-8.5026	< 2e-16 **
June	0.4854	0.3022	1.6062	0.1082
March	0.6827	0.3010	2.2681	0.0233 *
May	0.9822	0.3004	3.2696	0.0011 **

** significant at the 1% probability level
Null deviance: 53.766 on 25 degrees of freedom
Residual deviance: 41.947 on 22 degrees of freedom
AIC: 102.43

Logistic regression output for *Neoehrlichia mikurensis* prevalence.

	Estimate	Standard error	z value	Pr(>|z|)
Intercept	-3.0265	0.3620	-8.3605	< 2e-16 **
June	1.3765	0.4149	3.3177	0.0009 **
March	0.4005	0.4781	0.8377	0.4021
May	1.6753	0.4177	4.0108	6.05e-05 **

** significant at the 1% probability level
Null deviance: 89.450 on 25 degrees of freedom
Residual deviance: 62.293 on 22 degrees of freedom
AIC: 109
