# Supplementary material for: Environmental hazard of tick-borne diseases in urban and peri-urban sites in an endemic area of eastern France
Source: Parasite. 2026 Jul 29;33:40. doi: 10.1051/parasite/2026043 (PMC13427044; doi:10.1051/parasite/2026043)
Supplement: Supplementary file 4 — Supplementary Table S1: Number of Ixodes ricinus nymphs collected at the sites from March to June 2024. Counts for each triplicate and total per site and month. [file parasite-33-40-s4.pdf]

Table S1: Number of *Ixodes ricinus* nymphs collected at the sites from March to June 2024.
Counts for each triplicate and total per site and month.
* No Data

	Number of *Ixodes ricinus* collected nymphs				
Month<br>Site	March	April	May	June	Total
1- Robertsau forest North	8-10-5<br>23	29-37-12<br>78	56-44-55<br>155	21-6-14<br>41	297
2- Pourtalès park North	2-0-1<br>3	1-4-2<br>7	10-5-4<br>19	7-19-24<br>50	79
3- Orangerie park Center	0-15-4<br>19	0-0-1<br>1	0-0-0<br>0	1-1-2<br>4	24
4- Botanical garden Center	*	0-3-0<br>3	1-2-2<br>5	1-0-1<br>2	10
5- Citadelle park Center	1-0-1<br>2	0-1-0<br>1	0-0-1<br>1	0-0-0<br>0	4
6- Schulmeister park - Center	0-0-0<br>0	0-0-0<br>0	0-0-1<br>1	0-0-0<br>0	1
7- Neudorf forest South	19-26-12<br>57	13-15-13<br>41	17-10-8<br>35	32-28-26<br>86	219
8- Rohrschollen forest - South	18-42-20<br>80	37-49-37<br>123	7-5-8<br>20	2-3-18<br>23	246
Total	184	254	236	206	880
