# Supplementary material for: Environmental hazard of tick-borne diseases in urban and peri-urban sites in an endemic area of eastern France
Source: Parasite. 2026 Jul 29;33:40. doi: 10.1051/parasite/2026043 (PMC13427044; doi:10.1051/parasite/2026043)
Supplement: Supplementary file 5 — Supplementary Table S2: Nymphal infection prevalence (NIP). Table S2a: Nymphal infection prevalence (NIP) for the 8 sites. Table S2b: Nymphal infection prevalence (NIP) for the 3 zones (ecosystems). Table S2c: Nymphal infection prevalence (NIP) for the 4 months. [file parasite-33-40-s5.pdf]

Tables S2: Nymphal infection prevalence (NIP)

	Robertsau	Pourtalès	Orangerie	Botanical	Citadelle	Schulmeister	Neudorf	Rohrschollen	Total
Infected nymphs	67	12	3	1	0	0	51	48	182
Non-infected	117	67	21	9	4	1	142	114	475
Total	184	79	24	10	4	1	193	162	657
**NIP %**	**36.4**	**15.2**	**12.5**	**10.0**	**/**	**/**	**26.4**	**29.6**	**27.7**
95% CI	29.5–43.9	8.4–25.4	3.3–33.5	0.5–45.9			20.5–33.3	22.9–37.4	24.3–31.3

Table S2a: Nymphal infection prevalence (NIP) for the 8 sites.
/: insufficient data

	North	City	South	Total
Infected nymphs	79	4	99	182
Non-infected	184	35	256	475
Total	263	39	355	657
**NIP %**	**30.0**	**10.3**	**27.9**	**27.7**
**95% CI**	**24.8–35.8**	**4.1–23.6**	**23.5–32.8**	**24.3–31.3**

Table S2b: Nymphal infection prevalence (NIP) for the 3 zones (ecosystems).

	March	April	May	June	Total
Infected nymphs	42	29	57	54	182
Non-infected	121	144	84	126	475
Total	163	173	141	180	657
**NIP %**	**25.8**	**16.8**	**40.4**	**30.0**	**27.7**
**95% CI**	**19.4–33.3**	**11.7–23.4**	**32.3–49.0**	**23.5–37.3**	**24.3–31.3**

Table S2c: Nymphal infection prevalence (NIP) for the 4 months.
