# Supplementary material for: Environmental hazard of tick-borne diseases in urban and peri-urban sites in an endemic area of eastern France
Source: Parasite. 2026 Jul 29;33:40. doi: 10.1051/parasite/2026043 (PMC13427044; doi:10.1051/parasite/2026043)
Supplement: Supplementary file 6 — Supplementary Table S3: Bacterial pathogens. Tested bacteria: BOR: Borrelia burgdorferi sensu lato, ANA: Anaplasma phagocytophilum, NEH: Neoehrlichia mikurensis. Table S3a: Pathogen abundance for each bacterium versus sites. Table S3b: Bacteria abundance versus zones (after grouping of sites). Table S3c: Bacteria abundance versus months. [file parasite-33-40-s6.pdf]

Table S3: Bacterial pathogens

Tested bacteria: BOR: *Borrelia burgdorferi* sensu lato, ANA: *Anaplasma phagocytophilum*, NEH: *Neoehrlichia mikurensis.*

	BOR	ANA	NEH	Analyzed nymphs	Infections	Infections %	95% CI
1- Robertsau forest	37	4	46	184	87	47.3	39.9–54.7
2- Pourtalès park	7	0	6	79	13	16.5	9.4–26.9
3- Orangerie park	3	0	0	24	3	12.5	3.3–33.5
4- Botanical garden	0	1	0	10	1	10.0	0.5–45.9
5- Citadelle park	0	0	0	4	0	0.0	/
6- Schulmeister park	0	0	0	1	0	0.0	/
7- Neudorf forest	41	3	16	193	60	31.1	24.7–38.2
8- Rohrschollen forest	39	2	9	162	50	30.9	24.0–38.7
Total	127	10	77	657	214*	32.6	29.0–36.3

Table S3a: Pathogen abundance for each bacterium *versus* sites.

/: insufficient data

* The total number of infections (214) out of a total of 657 nymphs analyzed is higher than the number of infected nymphs (182) due to the presence of 32 co-occurrences in some of the infected nymphs.

	BOR	ANA	NEH	Analyzed nymphs	Infections	Infections %	95% CI
North	44	4	52	263	100	38.0	32.2–44.2
City	3	1	0	39	4	10.2	3.3–25.2
South	80	5	25	355	110	31.0	26.3–36.1
Total	127	10	77	657	214	32.6	29.0–36.3

Table S3b: Bacteria abundance *versus* zones (after grouping of sites).

	BOR	ANA	NEH	Analyzed nymphs	Infections	Infections %	95% CI
March	35	3	11	163	49	30.1	23.3–37.8
April	21	5	8	173	34	19.7	14.2–26.6
May	38	1	29	141	68	48.2	39.8–56.8
June	33	1	29	180	63	35.0	28.9–42.5
Total	127	10	77	657	214	32.6	29.0–36.3

Table S3c: Bacteria abundance *versus* months.
